# Inherent Signals in Sequencing-Based Chromatin-ImmunoPrecipitation Control Libraries

**DOI:** 10.1371/journal.pone.0005241

**Published:** 2009-04-15

**Authors:** Vinsensius B. Vega, Edwin Cheung, Nallasivam Palanisamy, Wing-Kin Sung

**Affiliations:** 1 Computational and Mathematical Biology Group, Genome Institute of Singapore, Singapore, Singapore; 2 Cancer Biology and Pharmacology Group, Genome Institute of Singapore, Singapore, Singapore; 3 Michigan Center for Translational Pathology, University of Michigan Health System, Ann Arbor, Michigan, United States of America; Georgia Institute of Technology, United States of America

## Abstract

**Background:**

The growth of sequencing-based Chromatin Immuno-Precipitation studies call for a more in-depth understanding of the nature of the technology and of the resultant data to reduce false positives and false negatives. Control libraries are typically constructed to complement such studies in order to mitigate the effect of systematic biases that might be present in the data. In this study, we explored multiple control libraries to obtain better understanding of what they truly represent.

**Methodology:**

First, we analyzed the genome-wide profiles of various sequencing-based libraries at a low resolution of 1 Mbp, and compared them with each other as well as against aCGH data. We found that copy number plays a major influence in both ChIP-enriched as well as control libraries. Following that, we inspected the repeat regions to assess the extent of mapping bias. Next, significantly tag-rich 5 kbp regions were identified and they were associated with various genomic landmarks. For instance, we discovered that gene boundaries were surprisingly enriched with sequenced tags. Further, profiles between different cell types were noticeably distinct although the cell types were somewhat related and similar.

**Conclusions:**

We found that control libraries bear traces of systematic biases. The biases can be attributed to genomic copy number, inherent sequencing bias, plausible mapping ambiguity, and cell-type specific chromatin structure. Our results suggest careful analysis of control libraries can reveal promising biological insights.

## Introduction

Sequencing-based Chromatin-Immunoprecipitation (ChIP) study has been rapidly gaining traction. Introduced around late 2004 with ChIP-SACO [1], it is currently fast becoming the mainstream and definitive assays for studying transcription factor binding on a genome-wide scale. Development of next generation sequencing platforms further enabled researchers to sequence deeper and to develop various interesting techniques (e.g. ChIP-SACO [1], ChIP-PET [2], ChIP-STAGE [3], ChIP-Seq [4]). The goal of sequencing-based ChIP is to identify locations in the genome where TF-DNA interactions mostly likely occur. Such locations are expected to be enriched with the sequenced fragments. This is challenging due to the vast number of unspecific fragments sequenced along with the ChIP-enriched ones.

Many interesting techniques proposed thus far have been successfully applied to a host of high-throughput sequencing ChIP (htsChIP) data. We can loosely classify these techniques into (i) those that uses single htsChIP library solely (e.g. fragment clustering [1], [2], Monte-Carlo simulations [2], analytical distributions [3], [5], adaptive thresholding [5]) and (ii) those that leverage their analyses with control (or sometimes called background or input) libraries [4], [6]. Clearly, the presence of a control library facilitates better approximations of the profile of unspecific precipitations and thus gives a better filtering of the false positive enrichments.

Despite the importance of control libraries, they have received little attention. Their behaviors and characteristics are typically assumed, without sufficient prior investigation. Control libraries are primarily used to identify and/or negate systematic biases that are present in the ChIP library. It is thus important to understand those biases. We argue that the sources of these biases can be broadly categorized into four groups: (a) genomic copy number variations, (b) mapping bias, (c) sequencing bias, and (d) chromatin and/or experimental bias. This study intends to explore the extent of these systematic biases.

## Results

### Low Resolution Profile of Various ChIP Data Reflects the Underlying Genomic Copy Number

**Figure 1:**
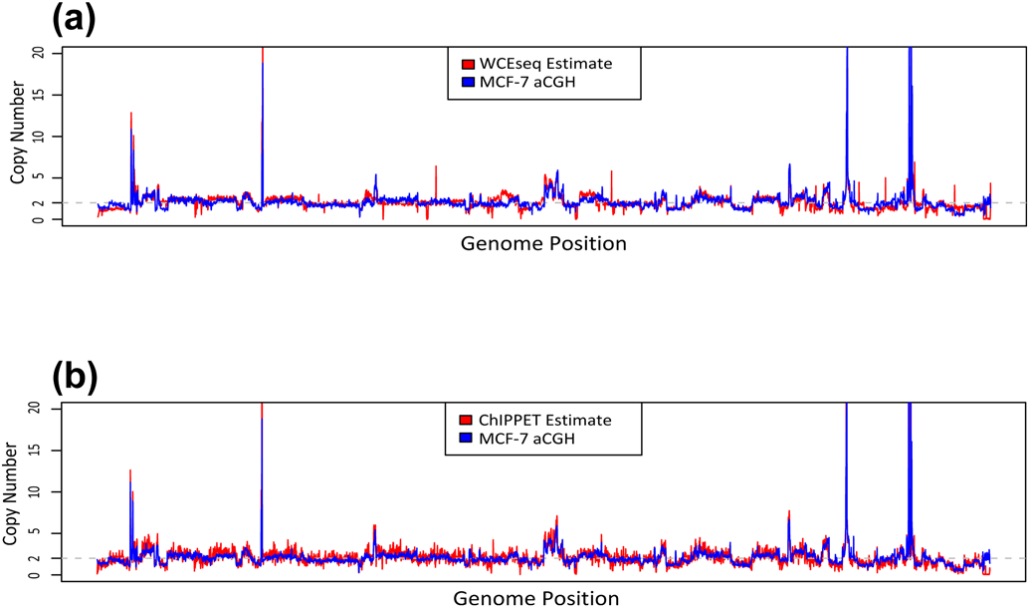
Whole cell extract sequencing (WCEseq) libraries are biased by genomic copy. The genome-wide copy number of MCF-7 (obtained from array CGH) at 1 Mbp resolution is contrasted to estimations made from (a) a WCEseq library and (b) ER ChIP-enriched library, sorted in chromosomal order. The high correlation (Pearson's r = 0.875) between WCEseq estimate and actual aCGH readout indicates coarse-scale profile of WCEseq library is dominantly shaped by copy number variations. Inherent effect of copy number variations also strongly affect ChIP-enriched library (Pearson's r = 0.673).

To investigate how much genomic copy number influence the control library, an in-house array CGH data (unpublished data – N.P.) of the MCF-7 cells was used as the benchmark for copy number variations in MCF-7. A whole cell extract library was also generated from MCF-7 and followed by direct ultra high-throughput sequencing using Solexa Genome Analyzer platform. Using Equation (1) and 1 Mbp sliding window (see Materials and Methods), we estimated the genome-wide copy number of MCF-7 based on the whole cell extract (WCEseq) library. As a comparison, we also took ChIP-enriched library (ER ChIP-PET [7]) and similarly estimated the genome-wide copy number using a signal-filtering approach and Equation (2). The copy number estimated from WCEseq library matched the array CGH readout very well (Pearson's r = 0.875, Fig. 1a). Interestingly, the estimate from ER ChIP enriched library agreed with the aCGH reasonably well too (Pearson's r = 0.673, see Fig. 1b).

**Figure 2:**
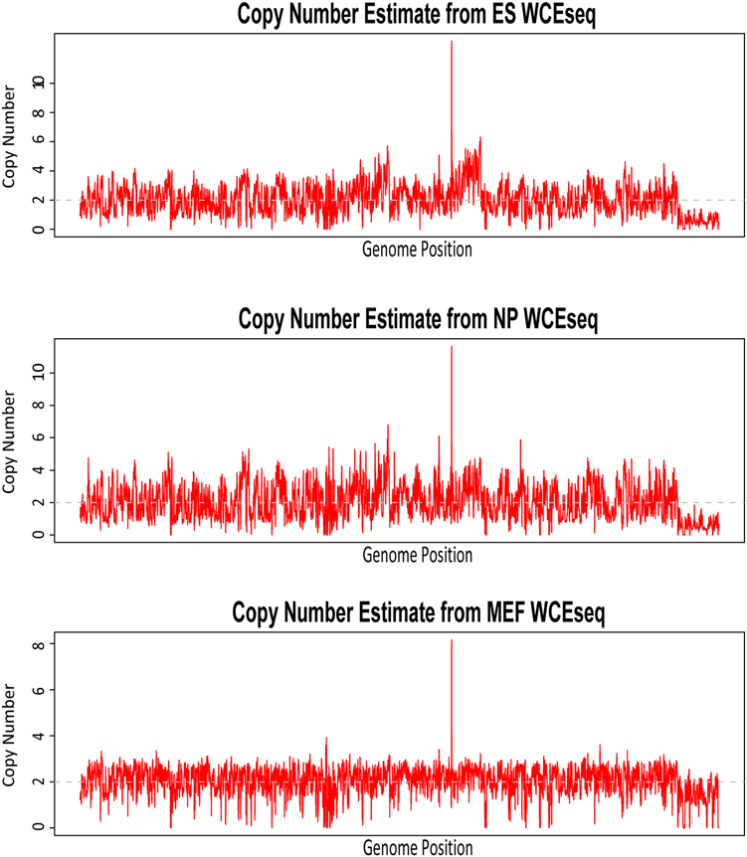
Comparison of genome-wide copy number from three mouse cell types (ES, NP, and MEF), sorted in chromosomal order. Although copy number wise, they were highly similar (Pearson's r>0.74 for all pairings) as expected, the exclusively high correlation (Pearson's r = 0.946) between ES and NP reflected their relationship at sample preparation level [8], [9].

Similar analyses were also performed using three mouse WCEseq libraries published by Mikkelsen *et al.* [8] which were generated from embryonic stem (ES), neural progenitor (NP), and embryonic fibroblasts (MEF) cells. Although the copy number estimates across these three libraries are generally similar (Pearson's r>0.74 for all pairings, Table 1), some differences were still apparent (Fig. 2). The correlation between that of ES and NP was unexpectedly high at almost 0.95, while the correlation between MEF and the other two libraries was about 0.75 on average. Although the copy numbers of these three cell types are expected to be very similar, the perceptible difference could be due to other reasons. One potential explanation could be due to how the libraries were generated. For example, the NP cells were derived from the ES, while the MEF was obtained independently [8], [9].

**Table 1:** Pairwise correlation of copy number estimates from three mouse WCEseq libraries.

Correlation	ES	NPC	MEF
**ES**	1	0.9464359	0.7546334
**NPC**	0.9464359	1	0.7428463
**MEF**	0.7546334	0.7428463	1

The genome-wide copy number for each cell type was estimated using the whole cell extract (WCEseq) library, based on Equation (1). The estimation was made based on 1 Mbp windows staggered by 500 kbp overlap.

### Effect of Tag Mapping Bias

**Figure 3:**
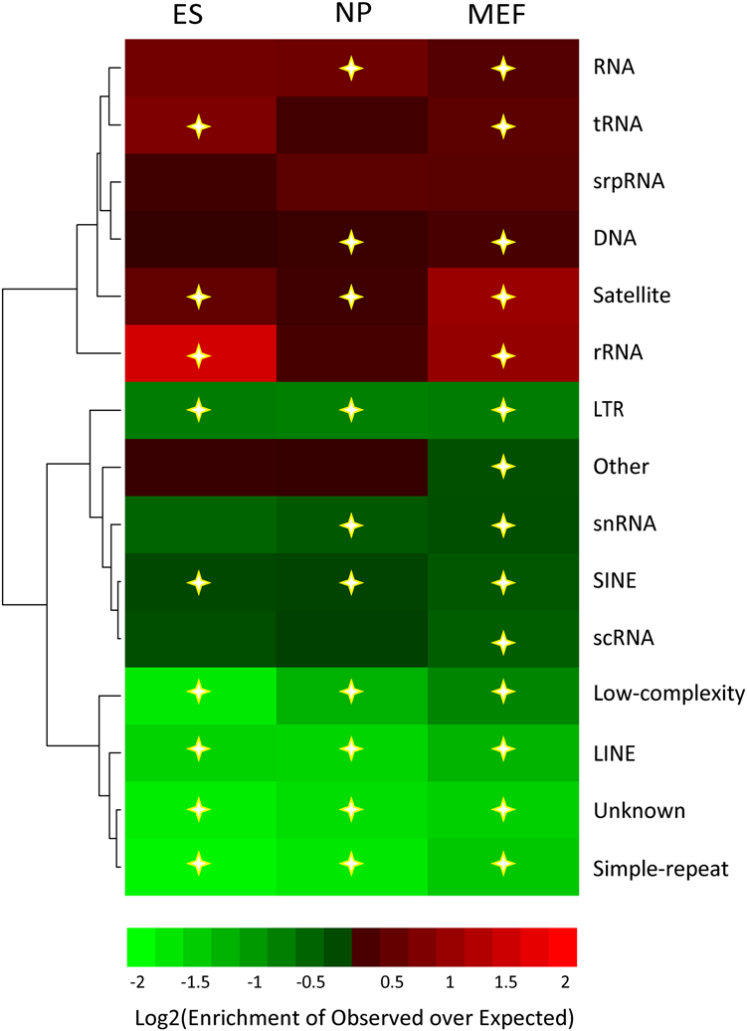
Mapping bias was apparent within repeat regions. Tag overabundance and paucity in the three mouse WCEseq libraries across various repeat classes, illustrating the biases due to mapping problems. Statistically significant deviations from random expectation (p-value<1e-3) were marked with stars.

Another likely source for systematic bias lies in the mapping procedures. For the purpose of assessing this bias, we used the repeat regions as a surrogate for heavily biased regions. We found that a number of repeat classes were significantly enriched (p<1e-3) for WCEseq tags, while some were unexpectedly depleted of tags (Fig. 3). The depleted region could be ascribed to mapping ambiguities in these repeats which resulted in the removal of these multiply mapped tags, as typically only uniquely mapped tags are retained. Satellite regions were found to be enriched in all the three WCE libraries. This was not unexpected as satellites have been previously reported to be unduly enriched in tags from ChIP-enriched libraries as well [10], marked by conspicuous spikes in otherwise flat genomic segments.

### Fine Resolution Oscillations are Correlated to Genomic Landmarks

Next, we examined the tag density distribution across the genome. From this analysis, we noticed that some of the spikes did not fall into any repeat regions. This led us to ask the following questions: How many significantly deviating spikes are there in a typical WCEseq library? Could they be all explained by Satellite or other repeat? Or are they coming from other genomic features? To answer this, we took the mouse WCEseq libraries and analyzed them at 5 kbp resolution. For each 5 kbp non-overlapping window, a p-value was computed for tag enrichment within the 5 kbp window assuming random uniform distribution of tags found in the overarching 1.5 Mbp region. Even after FDR-adjustment of multiple hypotheses [11], a considerable number of 5 kbp windows were enriched with tags (see Table 2). As expected, some of these tag-dense regions were artifacts from Satellite repeats. Interestingly, however, these tag-dense regions were much more significantly associated with a number of other genomic landmarks, namely Transcription Start Sites (TSS), Transcription End Sites (TES), and intragenic regions.

**Table 2:** Distribution of significantly enriched 5 kbp regions.

WCEseq Library	Significantly Dense 5 kbp Regions				
	Total	With TSS (Total: 20240)	With TES (Total: 21020)	Intragenic (Total: 182328)	With Satellite (Total: 3203)
ES	29	2 (6.9%)	2 (6.9%)	12 (41.38%)	11*** (37.93%)
NP	4334	1434*** (33.09%)	367*** (8.47%)	3825*** (88.26%)	55** (1.27%)
MEF	1403	1036*** (73.84%)	179*** (12.76%)	1186*** (84.53%)	38** (2.71%)

The significantly dense (FDR adjusted p-value<1e-4) 5 kbp regions (510,351 regions in total) across three WCEseq libraries were overlapped with gene boundaries (Transcription Start Sites and Transcription End Sites) annotation based on UCSC knownGene database for mm8 and tested for association using 1-tailed Fisher's Exact Test. An overlap with Satellite repeats was also done for comparison. The 5 kbp dense regions are significantly associated to genes and genes boundaries. (Notes: * = p<1e-3 ; ** = p<1e-5 ; *** = p<1e-16 ).

**Figure 4:**
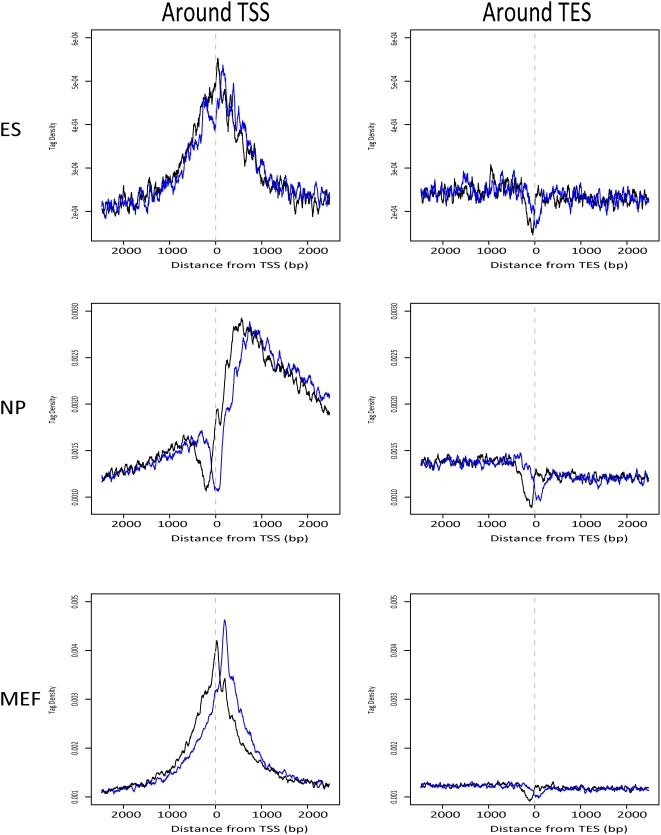
Chromatin bias in WCEseq library was evident along gene boundaries. Tag density (50 bp average) profiles around transcription start sites (TSS) and transcription end sites (TES) across three mouse WCEseq libraries. The black and blue curves denote density of tags mapped on the sense and antisense strands respectively.

In all the mouse WCEseq libraries used in this study, the TSS was correlated with a sharp spike of tag population (Fig. 4), however, the exact shape of the spike was library dependent. Tags in the WCEseq of ES and MEF peaked around the TSS, while tags in the NP WCEseq showed a dip at the TSS followed by a sharp increase around 500–700 bp downstream of the TSS. The peak enrichments ranged around 2.5, 2.75, and 4 times in NP, ES, and MEF WCEseq libraries respectively. In the NP WCEseq library, the peak was preceded by a steady upward trend upstream of the TSS followed by a gradual decline after the sharp jump downstream of TSS (Fig 4, middle left panel). In contrast, the tag density surrounding TES exhibited a punctuated profile of tags right at the TES (Fig 4, right panels). In ES WCEseq, the tag density at TES dropped by around a third of the density in the TES downstream regions, while both NP and MEF WCEseq experienced around 25% drop at the TES.

The dense 5 kbp regions were also pervasive among intragenic regions. Around 88.26% of the significantly dense 5 kbp regions of the NP WCEseq library were found to be associated with intragenic regions (Table 2). This observation was recapitulated in Figure 4 where the tag density at 2500 bp downstream of the TSS is still roughly 55% higher than that at 2500 bp upstream of the TSS in the NP WCE. A closer inspection of the density profiles surrounding the TSS in ES and MEF WCEseq libraries also revealed that the tag density in downstream regions of TSS, i.e. within intragenic regions, was more elevated compared to the promoter region, albeit only by about 12%, suggesting that gene bodies contain higher tag density. This trend was also observed around the TES, where the tag density upstream of the TES was generally higher compared to downstream of the TES, although only by approximately 8.5%, 12.8%, and 6.6% for ES, NP, and MEF WCEseq libraries respectively. From these observations, one might postulate a model where WCE fragments are accumulated significantly at the start of a gene region, followed by above than average density in the gene body, suddenly depleted at the end of the gene, and then leveling off to average density downstream of the gene (Supplementary Figure S3). Using an approximate of this model, we found on average 50% to 65% of genes corroborated this model (Supplementary Table S1).

### Tag Densities of Expressed and Non-Expressed Genes are Distinct

**Figure 5:**
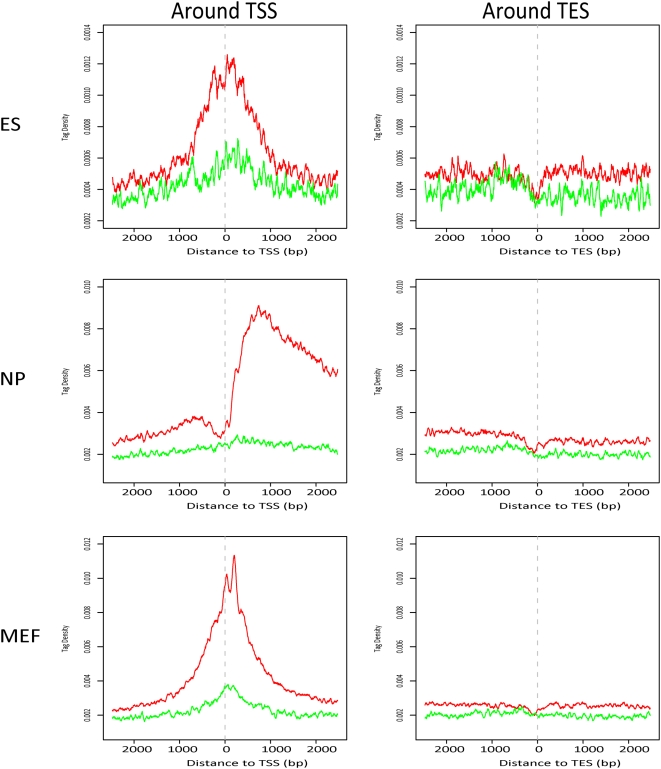
Expression levels of genes were correlated with tag density in WCEseq libraries. Density profiles (50 bp average) of tags (combined sense- and antisense-mapped) around TSS and TES of highly expressed (red) and lowly expressed (green) genes.

Using the accompanying expression data in [8], genes were grouped into high expressing and low expressing. We found that high-expressing genes exhibited a more pronounced profile of tag density around gene boundaries (Fig. 5), while low-expressing genes exhibited a more subdued contour, closer to genomic background. Overall, the TSS of high-expressing genes was populated by approximately four times more tags than the TSS of low-expressing genes, while regions around the TES of high-expressing genes contained ∼30% more tags than those of low-expressing genes.

### Effect of Sequencing Bias

It has been reported that the sequencing efficiency of next generation sequencers is influenced by the nucleic acid composition of the DNA fragment being sequenced [12], [13], where better sequencing efficiency is correlated with CG-rich sequences. This bias was generally mild among the three WCEseq libraries and was of a lesser degree compared to H3K4me3 ChIPseq (Supplementary Figure S4). Although the CG-bias was mild, we wondered whether the observed tag density pattern around TSS and TES could be explained solely by CG-dependent sequencing bias. The fact that the shape of tag density around TSS from WCEseq NP was markedly different from those of ES and MEF suggests that CG-bias could not have generated the observed patterns (Fig. 4). To investigate this more rigorously, we first formulated a model of high-throughput sequencing data generation which takes into account three primary influencing factors: (i) underlying fragment generation distribution, (ii) CG-dependent sequencing bias, and (iii) mapping bias (see Equation 3 in Materials and Methods). Assuming a null hypothesis of uniform fragment generation across the genome, we normalized the tag density profiles for CG-dependent sequencing bias (Equation 4). Since the mm8 genome is generally AT-rich, this null hypothesis has the effect of over dampening any real signal that happens to be CG-rich. Even so, we found that gene boundaries were still marked by distinct density profiles (Supplementary Figure S5) and high-expressing genes were more enriched with tags than low-expressing genes (Supplementary Figure S6).

## Discussions

### A Large Proportion of the Fragments are Noise Influenced by Genomic Copy Number and Other Biases

We started our analyses by comparing genome-wide profiles of various libraries at low 1 Mbp resolution. The fact that we could reasonably estimate the copy number using fragment density at low 1 Mbp resolution supports the assumption that a significant proportion of the fragments are random noise from the genome and that these random noise are predominantly influenced by the underlying genomic copy number. Consequently, this also supports the notion that WCEseq library should be able to negate bias from underlying chromosomal abundance (copy number). Having said that, though, copy number did not appear to be the sole component in influencing genome-wide profiles of WCEseq. When comparing three WCEseq libraries, which are from very similar and relatively normal genomes, we saw that they were not extraordinarily correlated even at low resolution. The observation suggested the presence of other biases. This was further confirmed by analyses at higher resolution, in which we found that tag-rich 5 kbp regions were non-randomly associated with repeats and gene boundaries (TSS and TES).

### Non-uniformity of Tags at Finer Scale Seems to be Driven by Chromatin Structure

From our analyses of localized spikes and dips around the TSS and TES, one might suspect that these features are primarily due to mapping bias. If this is the case, the three mouse WCEseq libraries should have roughly the same profile. However, we instead observed clearly distinct shapes of tag density at the TSS. Furthermore, the consistent phased profiles of sense and antisense tags (Fig. 4) suggested presence of well-positioned fragments that were recurrently sequenced. This phasing was similar to the phasing that marked well-positioned nucleosome [14]. Such phasing was not merely artifacts in tag-dense regions, as tag-dense satellite regions did not exhibit this profile (Supplementary Figure S1). Therefore, all these patterns are likely due to chromatin bias, and not mapping biases.

### The Signal Contained in WCEseq Appear to be Cell Type or Experiment Specific

All the evidence gathered thus far strongly suggests that WCEseq profile is cell-type specific. Since sequencing and mapping biases are expected to be similar among libraries of the same species, the cell-type specific signals should be coming from the other two sources of bias (i.e. copy number or chromatin/experiment bias); although it has to be noted that the degree of tag enrichment or scarcity in repeat regions (which are the archetypic regions with mapping bias) were not completely uniform among the mouse WCEseq libraries. Obviously, WCEseq profiles will be different if the different cell types have distinct copy number profiles. However, chromatin bias was apparent in WCEseq from ES, NP, and MEF cells, which are expected to be normal and non-amplified. Tag densities near gene boundaries were distinct in the three libraries and were correlated to the genes' expression levels. For example, only 8.63% of the significantly dense 5 kbp regions found in NP WCEseq library was also found to be significantly dense in MEF WCEseq library (Supplementary Figure S2a). Even among TSS-associated dense regions, only 20.6% of those found in NP WCEseq were common with those in MEF WCEseq (Supplementary Figure S2b). Beyond gene boundaries, we postulate that these tag dense and sparse regions also reflect other cell-type specific chromatin structures. For example, tag dense regions might generally correlate with open chromatin, which is in line with the suggestion in [15] that size-selection and sequencing might favor fragments from open chromatin regions.

Using primarily the WCE libraries, we have shown some extent of systematic biases attributed to genomic copy number variations, sequencing-and-mapping bias, as well as chromatin/experimental bias. Since the systematic biases present in the control library would influence the ChIP-enriched libraries as well, it is not inconceivable that more thorough analyses of the control library could potentially reduce false positive rate and false negative rate in binding sites identification, while concurrently provide insights into the underlying chromatin structure.

## Materials and Methods

### Datasets

This study made use of four whole cell extract sequencing data, which we call WCEseq. Three WCEseq libraries (from mouse ES, NP, and MEF cells) were obtained from a published work [8] and one (from human MCF-7 cells) was generated in-house. Tags were mapped into mm8 or hg18 accordingly. Only uniquely mapped tags were retained. The starting coordinate of the genome alignment were taken as the coordinate for the tag. Mapped tags were grouped into those mapped to the forward strand and those mapped to the reverse strand. Redundant tags in each group, defined as tags mapped to exactly the same genomic location, were removed. An additional ChIP-enriched library (ER ChIP-PET library [7] hg17-mapped) was also analyzed. Mouse ES, NP, and MEF H3K4me3-ChIPseq libraries [8] were used as a comparison in CG content bias analysis. Expression data [8] for the three mouse cells were employed to stratify genes based on expression level.

An array comparative genomic hybridization readout (using Agilent Human aCGH platform containing approximately 43,000 oligonucleotide features, based on hg17 assembly) was also obtained to measure the genomic copy number of MCF-7.

### Genomic Copy Number Estimation

The following method was used to generate genomic copy number estimation using WCEseq library. With the assumption that other biases are minimal and should not greatly affect the distribution of the tags, the genomic copy number of a given region can be estimated as:
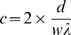
(1)where *c* is the estimated copy number, *d* is the number of tag counts within the region, *w* is the length of the region, and *λ* is the expected number of tags per base pair computed as the total number of tags in the library divided by the total gap-less genome length.

Genomic copy number estimation from ChIP-PET data requires two fundamental steps. First, as the library contains both signal and noise fragments, we need to first be able to extract the noise part. For this we consider only singleton PETs [5] and reduce PET cluster into a single pseudo singleton PET. For a given region, the relationship between the number of composite singletons (true singletons+pseudo singletons) *d* and copy number *c* can be described using Equation 2 below:
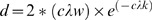
(2)


The first term of equation 2 denotes the amount of singletons expected had there be no overlapping of random PETs in a region, where *λ* is the expected number of tags per base pair computed locally for each region being considered. The second term denotes the fraction of random PETs expected not to overlap with other fragments [5].

In our analysis, we used sliding windows (1 Mbp in size, 500 kbp step size) to compute the average copy number from MCF-7 ER ChIP-PET, MCF-7 WCEseq, as well as from the three mouse WCEseq libraries. The same sliding windows were used in averaging the copy number readouts from the MCF-7 aCGH data, which was used as the benchmark in the MCF-7 study. Pearson's correlation was employed to assess the signal concordance within these windows among every pair of libraries. Comparison of MCF-7 aCGH and MCF-7 ER ChIP-PET was done based on hg17. To compare the aCGH data to WCEseq estimate, we first converted the aCGH data into hg18 assembly using the liftOver tool of UCSC Genome Browser.

### Tag Density Calculation and Normalization

Tag densities computed in our study were based on 50 bp averaging and normalized against the total number of regions inspected. Tags mapped to sense strand and tags mapped to antisense strand were considered separately in Figure 4. This allowed us to observe a consistent shift between them, indicating presence of consistent and well-positioned fragments with respect to the reference points (i.e. TSS and TES). Such consistent shift was not observed in the equally tag-rich satellite repeats, where the middle of repeat instances was used as the reference point (Supplementary Figure S1). We further grouped the genes based on their average expression level (Fig. 5). Probes were mapped to genes based on UCSC Genome Browser database [16]. Genes associated to the highest 25% expression readouts were classified as highly expressed and those associated to the lowest 25% were deemed as lowly expressed genes. Chromosomes X, Y, and M were ignored in this part of the study.

### Assessing Bias in Repeat Regions

As a proxy for mapping bias, we looked for irregularities in the number of tags mapped to different repeat classes. Repeat annotations were taken from UCSC Genome Browser database [16]. For each repeat class, the total number of tags found in its instances were counted and compared to the expected counts had the tags been randomly distributed across the genome. Figure 3 shows the enrichment and depletion of tags across repeat classes. Their significance was assessed using 1-tailed Binomial test. Those with p-value less than 1e-3 were considered statistically significant.

### Identification of Fine Scale Dense Regions

Having observed that copy number variation explains the coarse-scale profile of WCEseq libraries, we asked whether there exist finer-scale irregularities beyond what can be explained by copy number. To do this, we divided the genome into 5 kbp non-overlapping windows and assessed overabundance of tags while taking into account the local tag density within 1.5 Mbp window. For each window, we compute a p-value of tag overabundance using Poisson distribution as a null hypothesis, with the expected rate of tags based on the 1.5 Mbp window. After calculating the p-values for all 5 kbp windows, the p-values were corrected for multiple hypotheses using the FDR method [11]. Regions with adjusted p-value<1e-4 were deemed to be enriched. In this study we placed an emphasis on tag-rich regions and not tag-poor regions, as scarcity of tags could be affected by numerous other issues beyond the scope of this study.

The identified tag-rich 5 kbp regions were then associated with gene regions and boundaries (based on UCSC knownGene database [15]), as laid out in Table 2. As a positive control, amount of overlap with satellite repeats was also included. Significance of association was analyzed using 1-tailed Fisher's Exact Test. Interestingly, associations with gene body and boundaries were much more significant than association with satellite repeat. Association with TSS was exceptionally high. It in fact could explain most of the common dense regions found in both NP and MEF libraries (see Supplementary Figure S2).

### Testing the Model of Tag Density around Gene Regions

Detecting rises and drops of tag densities at specific locations in the genome using the ES, NP, and MEF WCEseq libraries was challenging, due to the low overall genome coverage of the library. To test the gene model illustrated in Supplementary Figure S3, we asked how many genes have higher tag density in the gene body compared to its upstream and downstream regions. Upstream and downstream regions of genes were defined as regions 2–5 kbp upstream of TSS and downstream of TES to avoid reduce signal overflow from the gene region and to guard for inaccuracies of the reported positions of TSS and TES. To avoid potential confusions, double counting, as well as ambiguities associated with long genes, we took forward-strand mapped genes found in the UCSC knownGene database, retained genes shorter than 100 kbp, and removed those that were overlapping with other genes in the retained list. The result is shown in Supplementary Table S1.

### A Generalized Model of Tag Generation

Let *x* be a position in the genome. Assuming a fixed fragment length, let 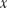 be the sequence of fragment associated with a tag at position *x* and 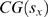 be proportion of C/G bases in 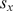. In this study we used the expected fragment length of 150 bp for defining 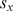. Let us also define binary variables 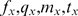, where 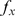 indicates whether fragments originated at position *x* are truly generated by the underlying experiment, 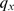 indicates whether tags at position *x* were successfully sequenced (or quantified), 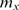 indicates whether tags at position *x* could be uniquely mapped, and 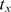 indicates whether a tag is actually observed at position *x*.

Following the above definitions, let 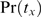 denotes the probability of observing a tag at position *x* in a given library. Clearly, 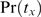 is directly proportional to the probability of fragments (which the tag represents) generated at position *x*, or 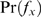. 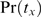 is also directly proportional to the probability that the tag being successfully sequenced, which are in turn dependent on the C/G composition of the fragment. This probability can be defined as 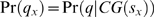. Finally, 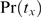 is directly proportional on whether the tag at position *x* could be mapped back with confidence to *x*, denoted as 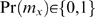. Taken together, we can model the tag generation as:
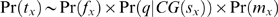
(3)The first term in the model is precisely the distribution that experimentalists wish to infer when constructing a sequencing library, while the second term and third term model the sequencing and mapping bias.

### Evaluating C+G Content Bias

We sought to roughly measure the bias that is correlated with the CG content. As a null hypothesis, libraries of simulated tags were constructed for tags of length 26 bp, 27 bp, and 29 bp, through random sampling of the genome sequences. Tags from H3K4me3 ChIPseq libraries were used as positive control. Comparing the resultant cumulative distributions, the WCEseq libraries were found to be relatively closer to the random tags compared to that of the H3K4me3 libraries (Supplementary Figure S4).

### Minimizing CG-dependent Sequencing Bias

One of the key goals of any high-throughput sequencing (hts) experiment is to infer the first factor, i.e. the underlying fragment distribution 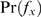. Part of the intention in generating control libraries is to use them to minimize the two biases. For this analysis, however, there was no further “control” for WCEseq libraries; although arguably non-crosslinked (naked) DNA libraries could be a good background control for WCEseq. The mapping bias can be controlled by characterizing the uniqueness of each genomic location. The CG-dependent sequencing bias is harder to mitigate and, under our model, is impossible to be normalized using only a single replicate data. Ideally, CG-dependent sequencing bias should be assessed through experimental means.

Given a hts library, we can measure the distribution of CG-content distribution of the DNA fragments associated with the observed tags, i.e. 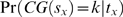. Expanding the term further:
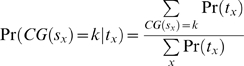
Expanding the numerator and defining 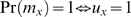 :
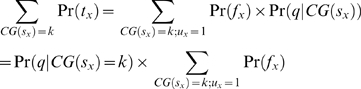
If 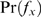 is indeed uniform across the entire genome, we can compute the CG-content distribution of uniquely mapped sequences as:
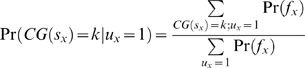
Combining the previous equations:
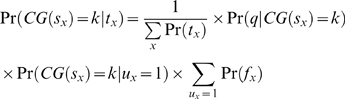
And thus
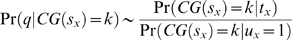
Therefore, if we assume that the fragment generation is uniform across the entire genome, we can normalize the CG-dependent sequencing bias as follow:
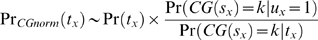
(4)


## Supporting Information

Table S1 Testing a model of gene profile bas on WCEseq tag density. A proxy test for tag density model around genes (Supplementary Figure S3) was carried out by comparing the tag density in gene body to the adjacent upstream and downstream regions. Upstream region was defined as the 2–5 kbp region 5′ upstream of TSS and downstream region was defined as the 2–5 kbp region 3′ downstream of TES. To avoid potential ambiguity, we considered only genes that were mapped to forward strand and were shorter than 100 kbp. Overlapping genes from this list were further removed.
(0.01 MB PDF)

Table S2 Sequencing depth of the libraries analyzed in this study
(0.01 MB PDF)

Figure S1 Comparative density profiles of tags mapped to forward strand (black lines) and reverse strand (blue lines) in a 5 kbp window centered around middle of Satellite repeats. As the enrichment of tags in Satellite repeats were likely to be resulted from mapping issues and other random noise, no well-positioned fragment was expected, resulted in closely correlating density profile of forward tags and reverse tags.
(0.09 MB PDF)

Figure S2 Comparison of 5 kbp tag-rich regions across WCEseq libraries. (a) A Venn diagram showing the tag-rich regions from the three Wcseq libraries. Regions from ES WCEseq library is negligible due to its shallow sequencing depth. Only 374 dense regions were found to be common in NP and MEF sets. It represented only 8.63% and 26.7% of tag-rich regions from NP and MEF libraries respectively. (b) Comparison of tag-rich regions that are associated with TSS. 296 TSS-associated tag rich regions were common, representing 20.6% and 28.6% of the total TSS-associated tag-rich regions found in the NP and MEF libraries. Common tag-rich regions of NP and MEF were mostly (296 of 374, or 79.1%) TSS-associated.
(0.02 MB PDF)

Figure S3 A schematic model of WCEseq fragments distribution across a typical gene, based on observations in Figures 4 and 5. Gene region is expected to be more fragment-rich than the immediate upstream and downstream regions, with the TSS marked with a substantial increase of fragment count and the TES punctuated with lower fragment count.
(0.01 MB PDF)

Figure S4 Cumulative distributions of tags based on their C+G content. Distributions of WCEseq tags (red curves) were relatively close to simulated tags (gray curves; based on 26 bp, 27 bp, and 29 bp tag lengths), indicating that sequence composition bias is relatively mild. As a comparison, similar curves generated from H3K4me3 ChIPseq tags were also drawn (green curves).
(0.06 MB PDF)

Figure S5 Tag density (50 bp average) profiles after CG-content normalization. The normalization assumed that each tag represents a 150 bp fragment, taking into account the tag direction. Each tag was reweighted such that the CG-content distribution of the fragments matched that of randomly sampled uniquely-mapped simulated tags. Shown above are profiles around transcription start sites (TSS) and transcription end sites (TES) across three mouse WCEseq libraries. The black and blue curves denote density of tags mapped on the sense and antisense strands respectively.
(0.12 MB PDF)

Figure S6 Expression levels of genes were correlated with CG-content normalized tag density in WCEseq libraries. Density profiles (50 bp average) of tags around TSS and TES of highly expressed (red) and lowly expressed (green) genes. The curves show combined density of sense- and antisense-mapped tags. Tags were reweighted based on the CG-content of the corresponding 150 bp fragments.
(0.13 MB PDF)
