# Supplementary material for: Inherent Signals in Sequencing-Based Chromatin-ImmunoPrecipitation Control Libraries
Source: PLoS One. 2009 Apr 15;4(4):e5241. doi: 10.1371/journal.pone.0005241 (PMC2666154; doi:10.1371/journal.pone.0005241)
Supplement: Table S1 — Testing a model of gene profile bas on WCEseq tag density. A proxy test for tag density model around genes (Supplementary Figure S3) was carried out by comparing the tag density in gene body to the adjacent upstream and downstream regions. Upstream region was defined as the 2–5 kbp region 5′ upstream of TSS and downstream region was defined as the 2–5 kbp region 3′ downstream of TES. To avoid potential ambiguity, we considered only genes that were mapped to forward strand and were shorter than 100 kbp. Overlapping genes from this list were further removed. (0.01 MB PDF) [file pone.0005241.s001.pdf]

Supplementary Table S1. Testing a model of gene profile bas on WCEseq tag density

WCEseq Library	Number of Genes		
	Total	Intragenic is more tag-rich than upstream	Intragenic is more tag-rich than downstream
ES	4205	2871 (68.28%)	2724 (64.78%)
NP	5124	3343 (65.24%)	3326 (64.91%)
MEF	5196	3315 (63.80%)	3216 (61.89%)

A proxy test for tag density model around genes (Supplementary Figure S3) was carried out by comparing the tag density in gene body to the adjacent upstream and downstream regions. Upstream region was defined as the 2-5kbp region 5’ upstream of TSS and downstream region was defined as the 2-5kbp region 3’ downstream of TES. To avoid potential ambiguity, we considered only genes that were mapped to forward strand and were shorter than 100kbp. Overlapping genes from this list were further removed.
