# Supplementary material for: Inherent Signals in Sequencing-Based Chromatin-ImmunoPrecipitation Control Libraries
Source: PLoS One. 2009 Apr 15;4(4):e5241. doi: 10.1371/journal.pone.0005241 (PMC2666154; doi:10.1371/journal.pone.0005241)
Supplement: Table S2 — Sequencing depth of the libraries analyzed in this study (0.01 MB PDF) [file pone.0005241.s002.pdf]

Supplementary Table S2. Sequencing depth of the libraries analyzed in this study

Library	Number of Uniquely Mapped Tags
MCF-7 ER ChIP-PET	136,152
MCF-7 WCEseq	5,347,233
ES WCEseq	715,231
NP WCEseq	4,074,894
MEF WCEseq	4,660,707
ES H3K4me3 ChIPseq	8,805,578
NP H3K4me3 ChIPseq	6,571,473
MEF H3K4me3 ChIPseq	11,239,010
