# Supplementary material for: Inherent Signals in Sequencing-Based Chromatin-ImmunoPrecipitation Control Libraries
Source: PLoS One. 2009 Apr 15;4(4):e5241. doi: 10.1371/journal.pone.0005241 (PMC2666154; doi:10.1371/journal.pone.0005241)
Supplement: Figure S1 — Comparative density profiles of tags mapped to forward strand (black lines) and reverse strand (blue lines) in a 5 kbp window centered around middle of Satellite repeats. As the enrichment of tags in Satellite repeats were likely to be resulted from mapping issues and other random noise, no well-positioned fragment was expected, resulted in closely correlating density profile of forward tags and reverse tags. (0.09 MB PDF) [file pone.0005241.s003.pdf]

Supplementary Figure S1

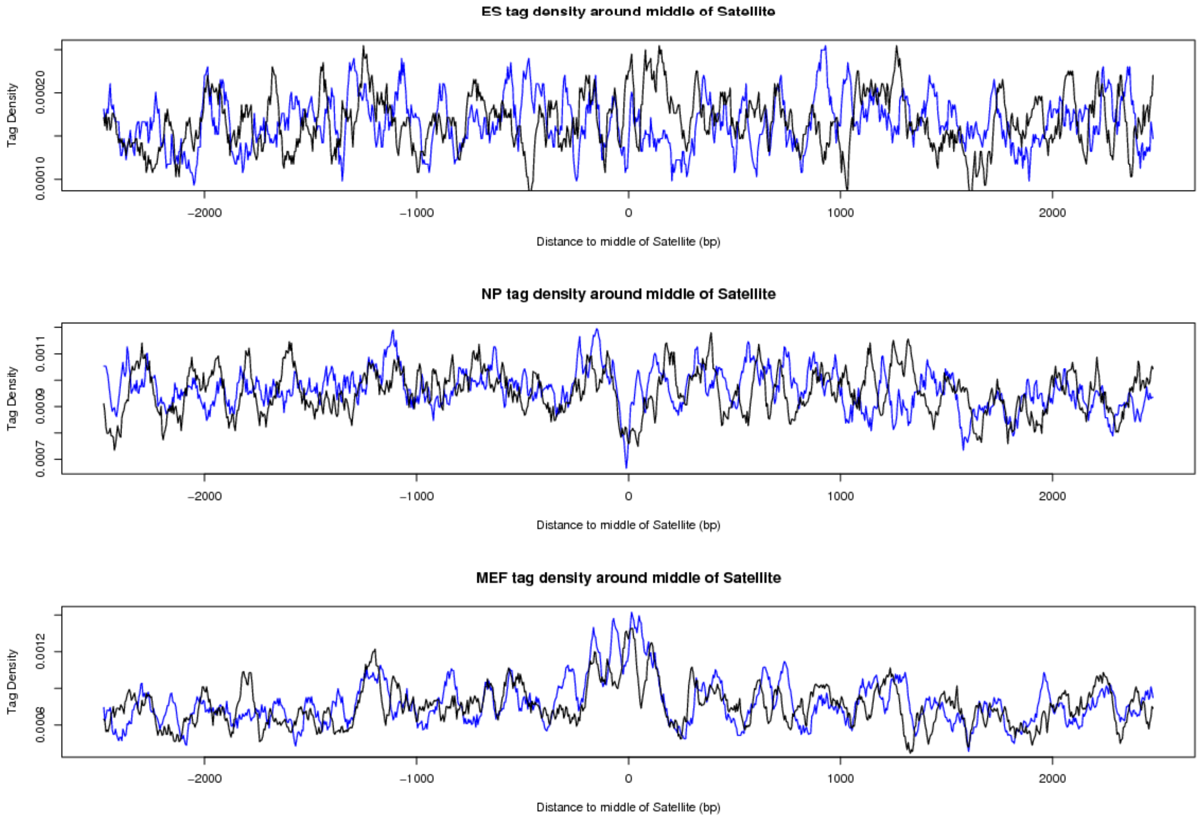

Supplementary Figure S1. Comparative density profiles of tags mapped to forward strand (black lines) and reverse strand (blue lines) in a 5kbp window centered around middle of Satellite repeats. As the enrichment of tags in Satellite repeats were likely to be resulted from mapping issues and other random noise, no well-positioned fragment was expected, resulted in closely correlating density profile of forward tags and reverse tags.
