# Supplementary material for: Inherent Signals in Sequencing-Based Chromatin-ImmunoPrecipitation Control Libraries
Source: PLoS One. 2009 Apr 15;4(4):e5241. doi: 10.1371/journal.pone.0005241 (PMC2666154; doi:10.1371/journal.pone.0005241)
Supplement: Figure S2 — Comparison of 5 kbp tag-rich regions across WCEseq libraries. (a) A Venn diagram showing the tag-rich regions from the three Wcseq libraries. Regions from ES WCEseq library is negligible due to its shallow sequencing depth. Only 374 dense regions were found to be common in NP and MEF sets. It represented only 8.63% and 26.7% of tag-rich regions from NP and MEF libraries respectively. (b) Comparison of tag-rich regions that are associated with TSS. 296 TSS-associated tag rich regions were common, representing 20.6% and 28.6% of the total TSS-associated tag-rich regions found in the NP and MEF libraries. Common tag-rich regions of NP and MEF were mostly (296 of 374, or 79.1%) TSS-associated. (0.02 MB PDF) [file pone.0005241.s004.pdf]

Supplementary Figure S2

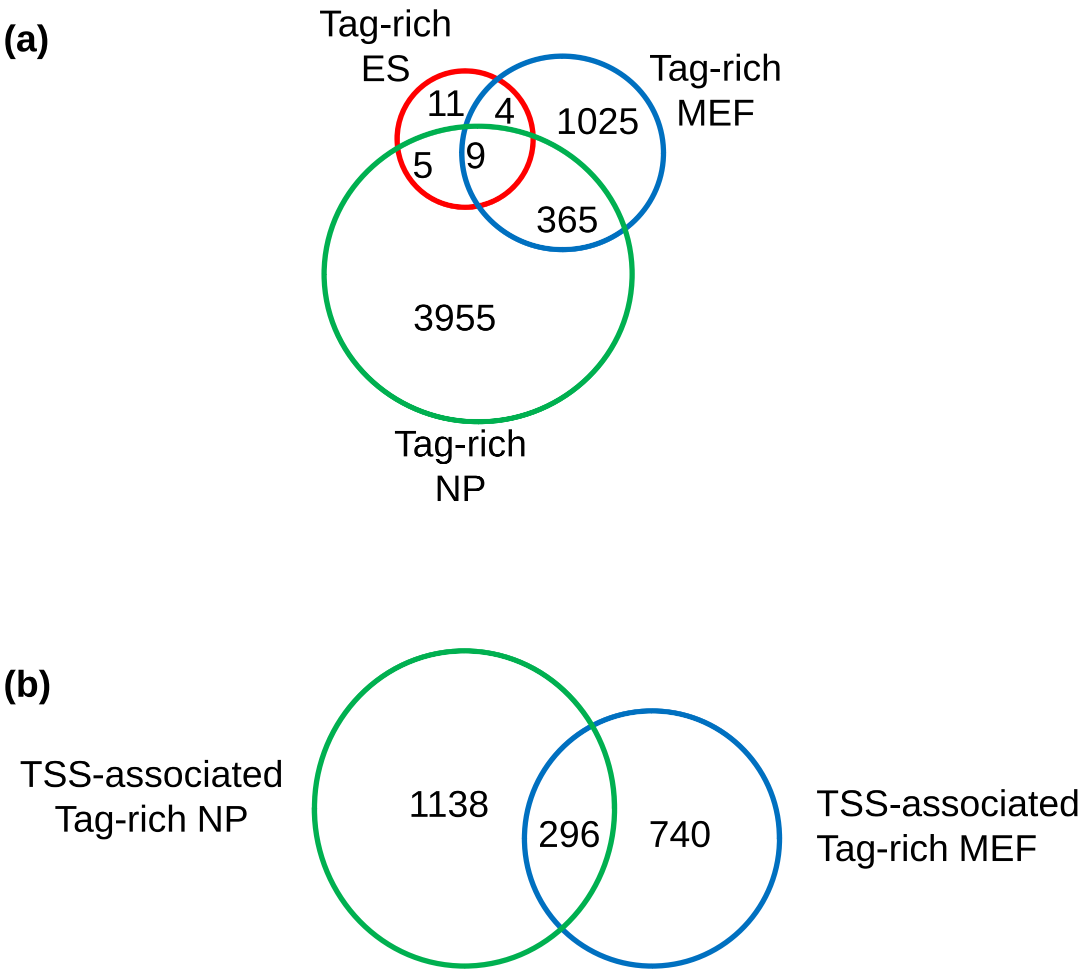

Supplementary Figure S2. Comparison of 5kbp tag-rich regions across WCEseq libraries. (a) A Venn diagram showing the tag-rich regions from the three Wcseq libraries. Regions from ES WCEseq library is negligible due to its shallow sequencing depth. Only 374 dense regions were found to be common in NP and MEF sets. It represented only 8.63% and 26.7% of tag-rich regions from NP and MEF libraries respectively. (b) Comparison of tag-rich regions that are associated with TSS. 296 TSS-associated tag rich regions were common, representing 20.6% and 28.6% of the total TSS-associated tag-rich regions found in the NP and MEF libraries. Common tag-rich regions of NP and MEF were mostly (296 of 374, or 79.1%) TSS-associated.
