# Supplementary material for: Inherent Signals in Sequencing-Based Chromatin-ImmunoPrecipitation Control Libraries
Source: PLoS One. 2009 Apr 15;4(4):e5241. doi: 10.1371/journal.pone.0005241 (PMC2666154; doi:10.1371/journal.pone.0005241)
Supplement: Figure S3 — A schematic model of WCEseq fragments distribution across a typical gene, based on observations in Figures 4 and 5. Gene region is expected to be more fragment-rich than the immediate upstream and downstream regions, with the TSS marked with a substantial increase of fragment count and the TES punctuated with lower fragment count. (0.01 MB PDF) [file pone.0005241.s005.pdf]

Supplementary Figure S3

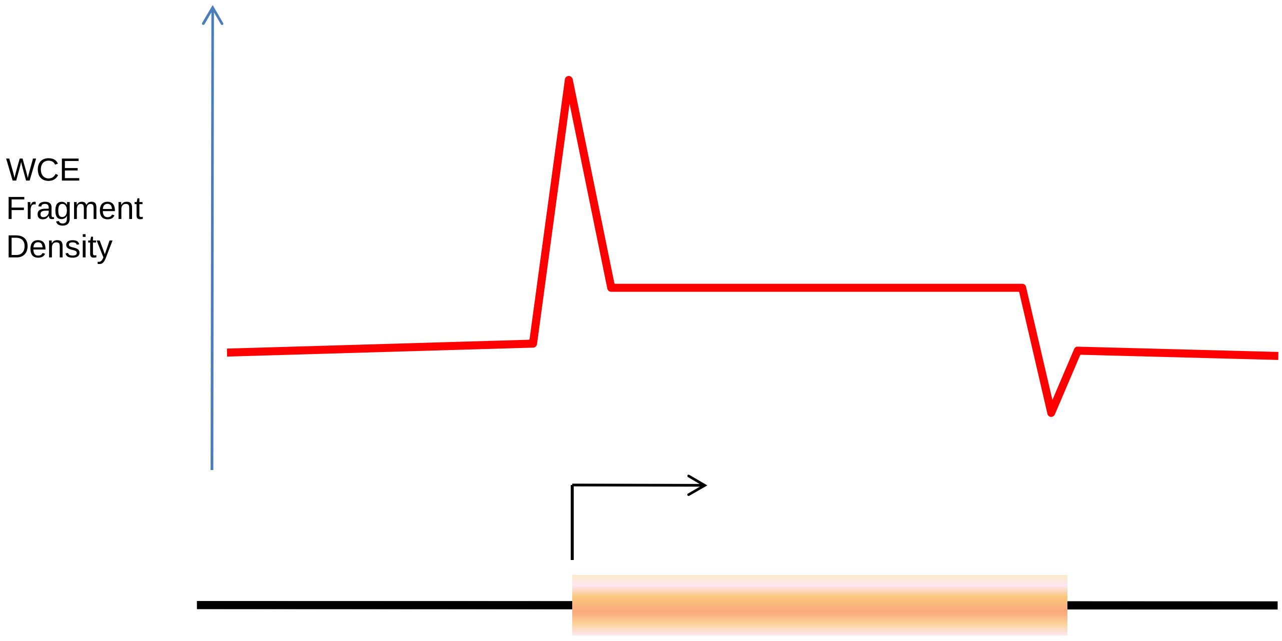

Supplementary Figure S3. A schematic model of WCEseq fragments distribution across a typical gene, based on observations in Figures 4 and 5. Gene region is expected to be more fragment-rich than the immediate upstream and downstream regions, with the TSS marked with a substantial increase of fragment count and the TES punctuated with lower fragment count.
