# Supplementary material for: Inherent Signals in Sequencing-Based Chromatin-ImmunoPrecipitation Control Libraries
Source: PLoS One. 2009 Apr 15;4(4):e5241. doi: 10.1371/journal.pone.0005241 (PMC2666154; doi:10.1371/journal.pone.0005241)
Supplement: Figure S4 — Cumulative distributions of tags based on their C+G content. Distributions of WCEseq tags (red curves) were relatively close to simulated tags (gray curves; based on 26 bp, 27 bp, and 29 bp tag lengths), indicating that sequence composition bias is relatively mild. As a comparison, similar curves generated from H3K4me3 ChIPseq tags were also drawn (green curves). (0.06 MB PDF) [file pone.0005241.s006.pdf]

Supplementary Figure S4

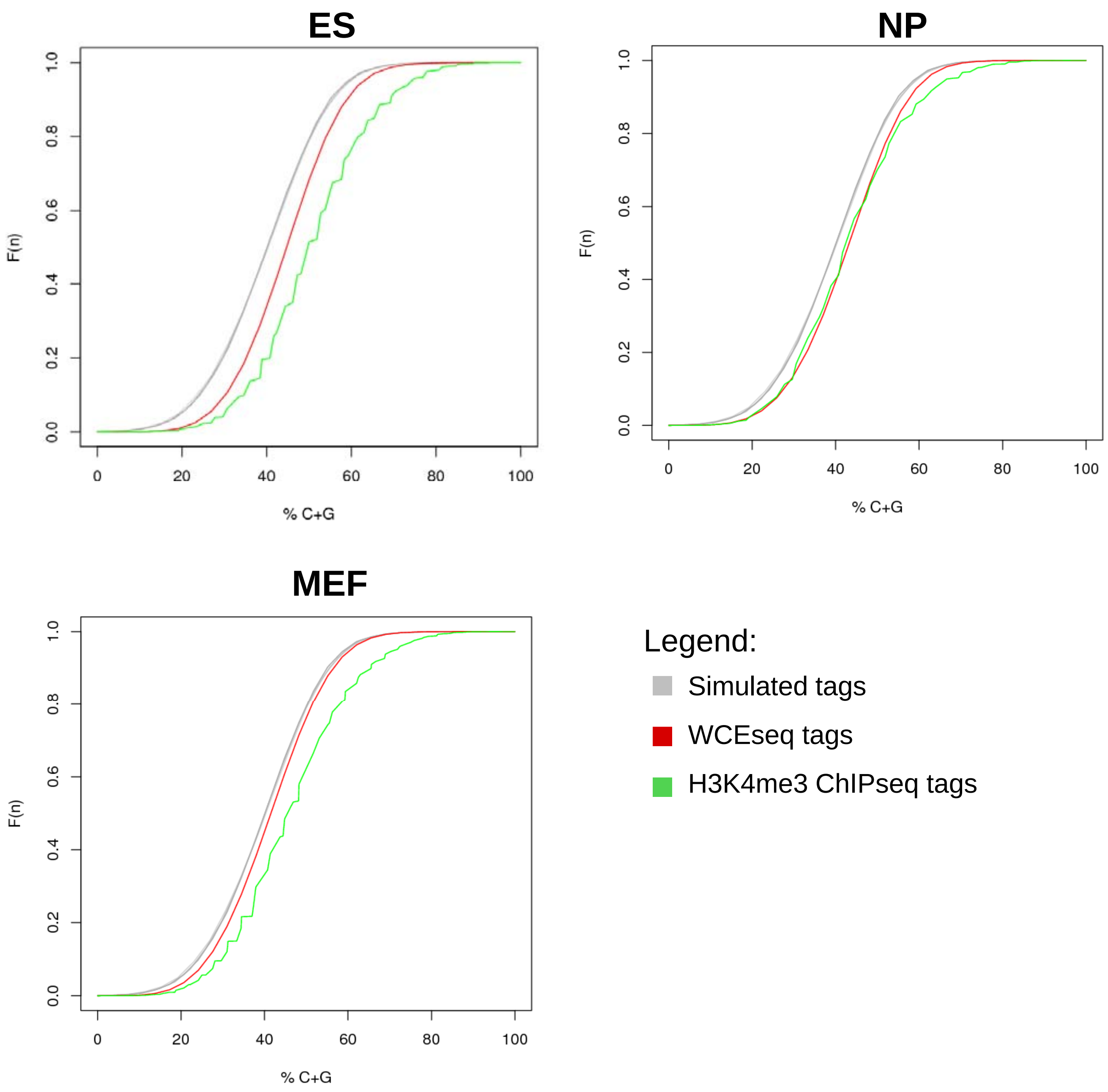

Supplementary Figure S4. Cumulative distributions of tags based on their C+G content. Distributions of WCEseq tags (red curves) were relatively close to simulated tags (gray curves; based on 26bp, 27bp, and 29bp tag lengths), indicating that sequence composition bias is relatively mild. As a comparison, similar curves generated from H3K4me3 ChIPseq tags were also drawn (green curves).
