# Supplementary material for: Inherent Signals in Sequencing-Based Chromatin-ImmunoPrecipitation Control Libraries
Source: PLoS One. 2009 Apr 15;4(4):e5241. doi: 10.1371/journal.pone.0005241 (PMC2666154; doi:10.1371/journal.pone.0005241)
Supplement: Figure S5 — Tag density (50 bp average) profiles after CG-content normalization. The normalization assumed that each tag represents a 150 bp fragment, taking into account the tag direction. Each tag was reweighted such that the CG-content distribution of the fragments matched that of randomly sampled uniquely-mapped simulated tags. Shown above are profiles around transcription start sites (TSS) and transcription end sites (TES) across three mouse WCEseq libraries. The black and blue curves denote density of tags mapped on the sense and antisense strands respectively. (0.12 MB PDF) [file pone.0005241.s007.pdf]

Supplementary Figure S5

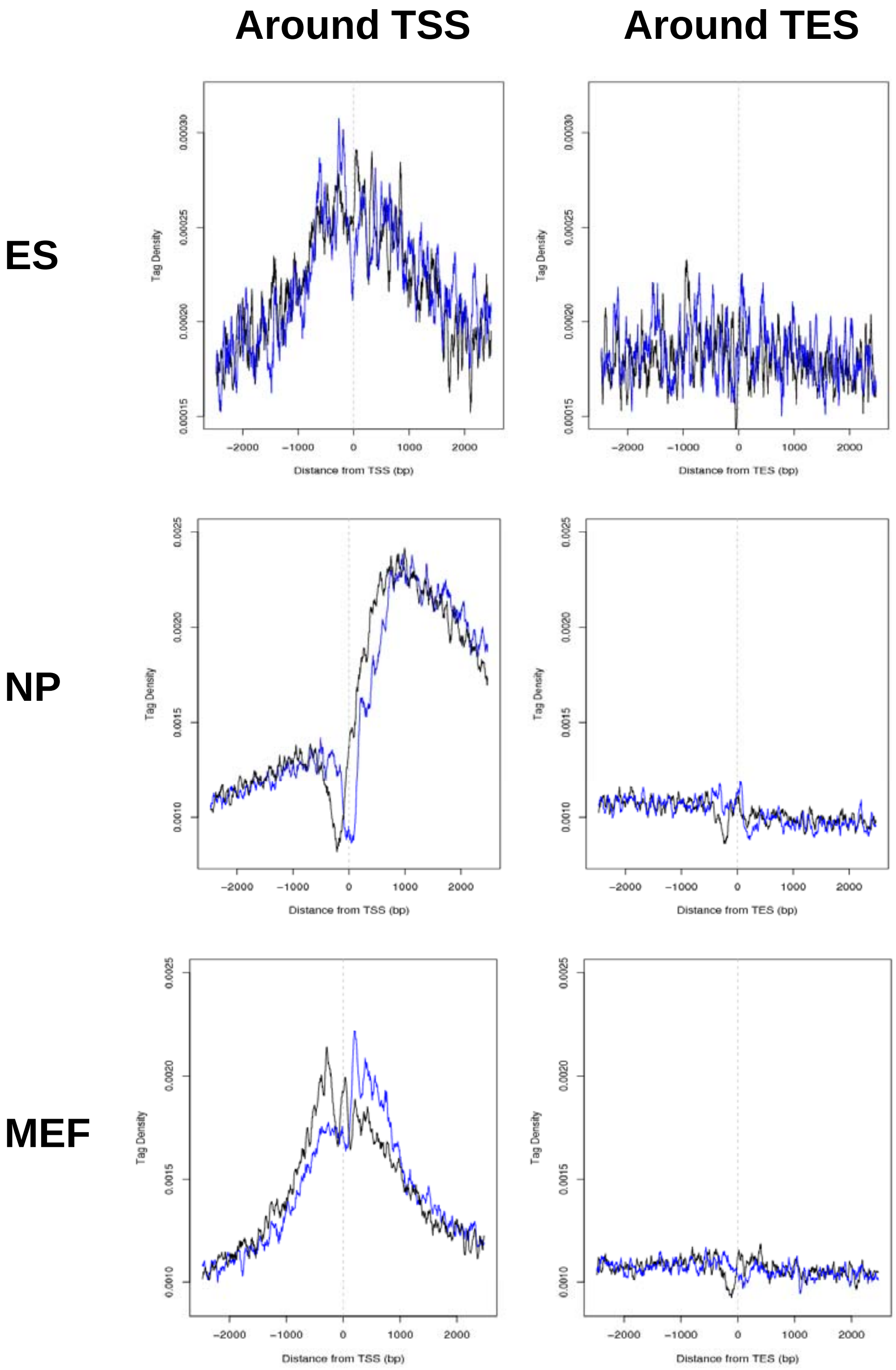

Supplementary Figure S5. Tag density (50bp average) profiles after CG-content normalization. The normalization assumed that each tag represents a 150bp fragment, taking into account the tag direction. Each tag was reweighted such that the CG-content distribution of the fragments matched that of randomly sampled uniquely-mapped simulated tags. Shown above are profiles around transcription start sites (TSS) and transcription end sites (TES) across three mouse WCEseq libraries. The black and blue curves denote density of tags mapped on the sense and antisense strands respectively.
