# Supplementary material for: Inherent Signals in Sequencing-Based Chromatin-ImmunoPrecipitation Control Libraries
Source: PLoS One. 2009 Apr 15;4(4):e5241. doi: 10.1371/journal.pone.0005241 (PMC2666154; doi:10.1371/journal.pone.0005241)
Supplement: Figure S6 — Expression levels of genes were correlated with CG-content normalized tag density in WCEseq libraries. Density profiles (50 bp average) of tags around TSS and TES of highly expressed (red) and lowly expressed (green) genes. The curves show combined density of sense- and antisense-mapped tags. Tags were reweighted based on the CG-content of the corresponding 150 bp fragments. (0.13 MB PDF) [file pone.0005241.s008.pdf]

Supplementary Figure S6

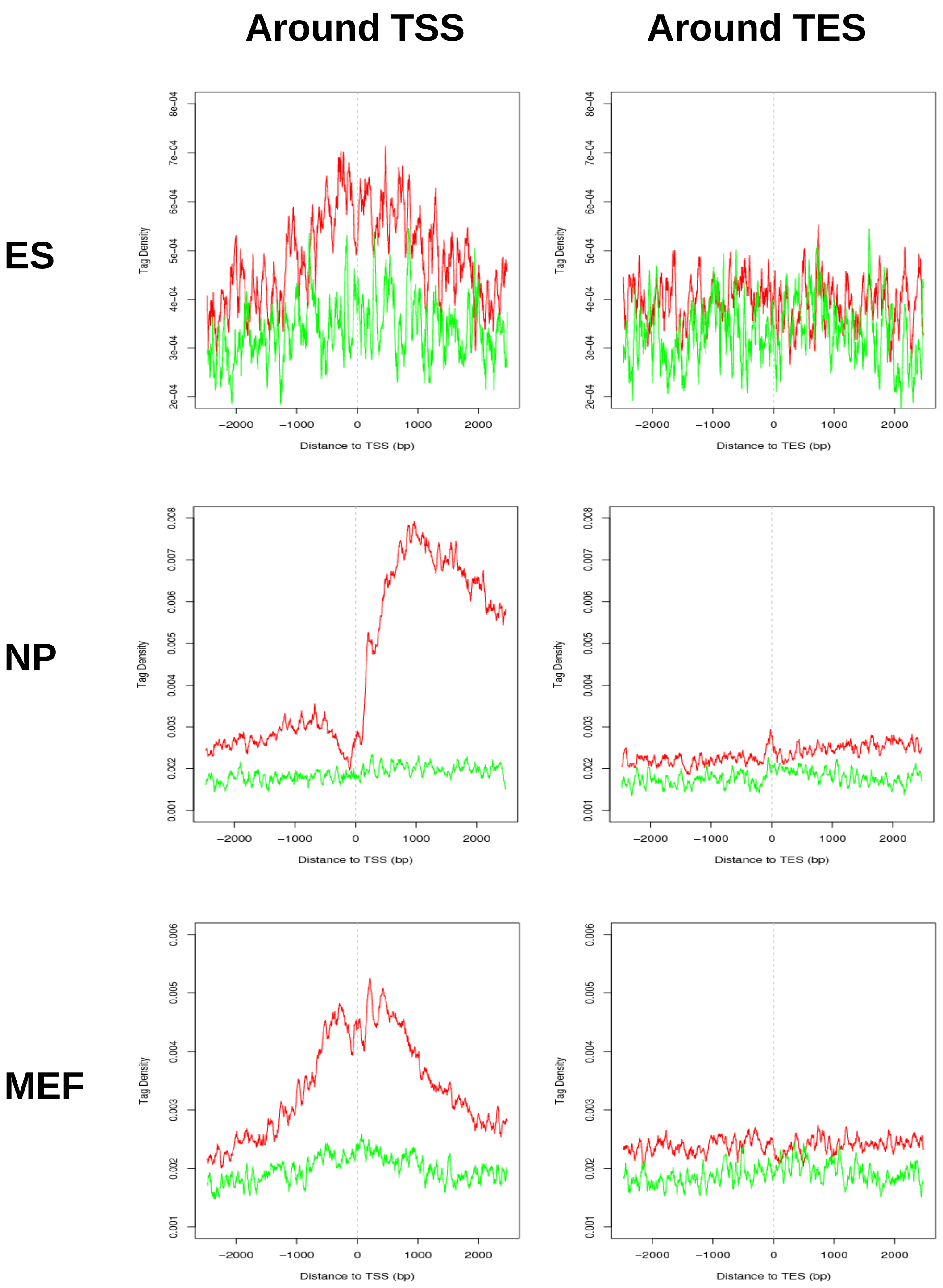

Supplementary Figure S6. Expression levels of genes were correlated with CG-content normalized tag density in WCEseq libraries. Density profiles (50bp average) of tags around TSS and TES of highly expressed (red) and lowly expressed (green) genes. The curves show combined density of sense- and antisense-mapped tags. Tags were reweighted based on the CG-content of the corresponding 150bp fragments.
